# The impact of hierarchical plateau on civil servants’ taking charge behavior: The role of work engagement and trait mindfulness

**DOI:** 10.1371/journal.pone.0315916

**Published:** 2024-12-17

**Authors:** Linsheng Xiao

**Affiliations:** Beijing Institute of Technology-Zhuhai, Business School, Zhuhai, Guangdong, China; Xi’an Jiaotong-Liverpool University, CHINA

## Abstract

Taking charge behavior of civil servants is important for enhancing the transformative capacity and governance efficacy of the public sector. This study is based on the conservation of resources theory and the job demands-resources model to explore the impact of hierarchical plateau on taking charge behavior of civil servants, including the mediating role of work engagement and the moderating effect of trait mindfulness. Through the analysis of a sample of 286 civil servants in Guangdong Province, China, the results show that hierarchical plateau has a negative effect on taking charge behavior of civil servants, with work engagement partially mediating the relationship between hierarchical plateau and taking charge behavior; trait mindfulness can alleviate the negative relationship between hierarchical plateau and work engagement. Furthermore, the mediating effect of work engagement is moderated by trait mindfulness, with higher levels of civil servants’ trait mindfulness weakening the indirect influence of hierarchical plateau on taking charge behavior through work engagement. The findings of this study deepen the understanding of the psychological mechanism of hierarchical plateau on the taking charge behavior.

## Introduction

Employees who take the initiative to make recommendations and implement changes are crucial to the long-term success of an organization. In recent years, both academia and practice have shown increasing interest in employee proactive behavior. As a form of proactive behavior, taking charge behavior refers to employees voluntarily and actively optimizing organizational structures, improving work patterns, and perfecting work processes to enhance the performance of individuals, teams, or organizations [1]. Against the backdrop of intensified international competition, sluggish economic growth, and fiscal austerity, taking charge behavior of civil servants is particularly important for enhancing the transformative capacity and governance efficacy of the public sector. On one hand, top-down reforms have led to the spread of "reform fatigue" among civil servants, increasing the resistance and cost of reforms [2]. On the other hand, many organizations have failed to achieve their intended reform goals and are unable to advance continuous systemic changes. Moreover, facing an increasingly complex work environment and diverse public demands, organizations find it difficult to predict the activities they desire or require civil servants to perform [3]. Therefore, the public sector needs employees who can implement ongoing bottom-up reforms and bring about constructive changes [4]. In other words, organizations expect employees not only to complete prescribed tasks according to job requirements but also to spontaneously change work methods, policies, and procedures to effectively address the severe challenges brought about by environmental changes [1,5]. Studies have shown that there is a significant difference between taking charge behavior and other behaviors such as voice behavior, change-supportive behaviors, and innovative behavior [1,6]. Compared to the aforementioned behaviors, taking charge behavior is more proactive because it is driven by a desire for continuous organizational improvement, actively challenging rather than passively adapting to the status quo, which is precisely the goal that the public sector strives to achieve in new environments [6]. Empirical studies have shown that employees’ taking charge behavior has a strong altruistic nature and can enhance organizational performance and competitiveness [7]. Given the importance of taking charge behavior to the public sector [3], it is necessary to fully explore the influencing factors and mechanisms behind civil servants’ taking charge behavior.

Research suggests that the factors influencing civil servants’ taking charge behavior, whether motivational or hindering, have not been systematically examined [8]. However, relatively speaking, existing literature has more frequently explored the motivational factors of civil servants’ taking charge behavior, including individual factors such as public service motivation [9,10], leadership factors such as transformational leadership [9] (Homberg et al., 2019) and leader’s taking charge behavior [8], job factors such as non-economic rewards [3], organizational systemic factors such as organizational support [9], and fault-tolerant mechanisms [11,12]. These factors are considered important prerequisites for taking charge behavior. In contrast, only a limited number of studies have examined the hindering factors of civil servants’ taking charge behavior, such as workload [3], and there is a noticeable lack of research on the relationship between the career development characteristics of the civil servant group and taking charge behavior. According to Max Weber, the public sector is a typical bureaucratic organization. The "pyramid" hierarchical structure is one of the core characteristics of bureaucratic organizations, and this structural feature determines that the public sector is always facing the issue of how to maintain the work vitality of civil servants within limited promotion spaces [13]. Promotion is the mechanism by which organizational members’ positions within the organization continuously rise along a pre-set hierarchical order [13]. Due to the multidimensionality of principal-agent relationships, the internal labor market of the public sector is difficult to provide competitive monetary incentives, thus promotion is the main way to motivate employees in the public sector [14]. Promotion is also one of the core goals pursued by organizational members, as it determines their economic income, social status, and organizational power [15]. However, as the pyramid moves towards the top with fewer positions available, more and more civil servants face a career "ceiling," leading to a sense of career plateauing and particularly severe stagnation in promotion.

Ference et al [16] first introduced the concept of career plateau, describing a period in an employee’s career development where they reach the peak of their career with very little chance of further promotion. Career plateaus mainly include dimensions such as hierarchical plateau and content plateau [17,18]. Considering that promotion is a primary method for motivating employees in the public sector, this study focuses on the impact of hierarchical plateau on civil servants’ taking charge behavior. Research highlights that promotion issues can affect the behavioral characteristics of officials, thereby also influencing the governance logic of the government [19]. Previous research have shown that hierarchical plateau can trigger civil servants’ work withdrawal behavior [19] and have a negative impact on job performance [20]. Studies based on corporate organizations have found that career plateaus not only weaken employees’ in-role performance [21] but also hinder employees’ organizational citizenship behaviors [22]. Based on the above understanding, it is expected that hierarchical plateau will have a negative impact on civil servants’ taking charge behavior.

If hierarchical plateau has an impact on civil servants’ taking charge behavior, what is the underlying mechanism at work? The conservation of resources theory (COR) posits that positions are important condition resources that have positive implications for an individual’s future life and work [23]. On the other hand, according to the job demands-resources (JD-R) model, career development opportunities related to personal growth, learning, and development are a type of job resource that can stimulate employees’ work motivation, increase work engagement, and thus positively affect work outcomes [24]. For civil servants, promotion is an important sign of career success. However, hierarchical plateau conveys that civil servants lack opportunities to promote their own growth, development, and achieve career aspirations; promotion stagnation also implies the loss of resources such as economic income, social status, and organizational power [15]. According to Hobfoll et al [25], resource loss can make individuals feel stressed and threatened, thereby inducing employees to treat work negatively, such as reducing their engagement to work [26]. Work engagement, as a high-energy motivational state, is considered an important condition for the formation of taking charge behavior [27,28]. Therefore, it is expected that hierarchical plateau will have a negative impact on taking charge behavior by reducing civil servants’ work engagement. Furthermore, to gain a more comprehensive understanding of the mechanism of hierarchical plateau, this study will also explore the boundary conditions under which hierarchical plateau affects civil servants’ taking charge behavior. Based on the COR theory, when facing stress or resource depletion, individuals will use existing resources to obtain new resources to reduce the net loss of resources, or increase resource investment to mitigate the impact of negative emotions [25]. Hobfoll [23] suggested personality traits to be important psychological resources for individuals that can buffer the adverse effects of stressors on individual psychology and behavior. With the rise of positive psychology, trait mindfulness, as a state-like trait variable with cross-temporal and cross-situational stability, has received widespread attention from scholars. Trait mindfulness refers to an individual’s ability to maintain awareness and attention on the present in an open, non-judgmental manner and is considered a positive personality trait [29]. Researchers have found that trait mindfulness can stimulate employees’ positive emotions [30], alleviate stress [31], and enhance employees’ cognitive functions and task performance [32]. Based on the above perspectives, this study uses trait mindfulness as a moderating variable to explore the boundary of the impact of hierarchical plateau on civil servants’ taking charge behavior, to test how the two jointly affect civil servants’ taking charge behavior.

To summarize, this study is based on the COR theory and the JD-R model to reveal the mechanism by which hierarchical plateau affect civil servants’ taking charge behavior and to explore the contingent effects of trait mindfulness on the above relationship. The theoretical contributions of this study are mainly as follows: first, this paper explores the negative impact of hierarchical plateau on civil servants’ taking charge behavior, expanding the research on the antecedents of civil servants’ taking charge behavior; moreover, this paper also responds to call of Yang et al [33] to strengthen the research on the relationship between career plateaus and employees’ extra-role behaviors. Second, this study reveals the mechanism by which hierarchical plateau affect civil servants’ taking charge behavior, providing new insights into the relationship between hierarchical plateau and taking charge behavior. Third, from the perspective of positive psychological traits, using trait mindfulness as a moderating variable, this study explores the boundary conditions that can weaken the negative impact of hierarchical plateau, providing a theoretical basis for organizations to carry out effective interventions.

## Theory and hypotheses

### Hierarchical plateau and taking charge behavior

Ference et al [16] defined career plateaus from the perspective of promotion. They believed that a career plateau refers to a certain stage in an individual’s career development, where the possibility of further promotion for the individual is very small. Milliman [18] referred to the career plateau defined by Ference et al. as a hierarchical plateau and also proposed another form of career plateau, namely job content plateau. A job content plateau refers to a situation where an individual has mastered all the knowledge and skills related to the job, and the work lacks a sense of challenge. A meta-analysis of career plateaus found that, as a typical source of work stress, career plateaus have a negative impact on employees’ attitudes and behaviors [33].

Promotion is one of the main factors for organizations to enhance employee work enthusiasm [34]. For civil servants, promotion is even more the core goal pursued in their career [15]. In hierarchical organizations, the higher the level of the position, the greater the organizational power exercised and the higher the benefits enjoyed. Therefore, promotion often comes with more resources, such as economic income, social status, organizational power, and even a broader social network [15]. Consequently, employees who feel that they lack the possibility of future promotion within the organizational hierarchy may view this stagnation as a threat to the valuable workplace resource benefits in the future [17]. According to the COR theory, individuals have a tendency to conserve, protect, and acquire resources. Therefore, both the threat of potential resource loss and actual resource loss, or when individuals have made efforts but the resources have not actually increased, can trigger stress and tension [23,25]. Hobfoll [23] further pointed out that the negative impact of resource loss on employee attitudes and behaviors may be greater than the positive impact of resource gains. On the other hand, organizational support is also considered an important work resource [37], but civil servants who perceive promotion stagnation may feel that they have not received recognition and support from the organization, which can trigger an individual’s negative reciprocity psychology [33]. Taking charge behavior, as an extra-role behavior that challenges the status quo, requires time and resources [3]. According to the COR theory and the JD-R model, abundant resources are the foundation for individuals’ positive work attitudes and proactivity. Sufficient resources are not only conducive to civil servants completing daily work and implementing in-role behaviors but also help to take charge behaviors that go beyond job responsibilities [35]. Therefore, when civil servants believe that their promotion goals cannot be achieved as expected and perceive a lack of organizational support, they are likely to experience negative emotions and negative cognitions, increasing the tendency for civil servants to avoid work both physically and mentally, such as slacking off and "holding a position without fulfilling duties" [19]. Hence, we hypothesize that:

H1: Hierarchical plateau has a significant negative predictive effect on taking charge behavior.

### The mediating role of work engagement

Work engagement refers to a positive, fulfilling, work-related state of mind that is characterized by vigor, dedication, and absorption [36]. Vigor is the willingness to invest effort and the sustained high levels of energy; dedication is the sense of significance, enthusiasm, inspiration, and pride in the job; absorption is the cognitive engagement and the feeling of being fully concentrated and happily engrossed in one’s work [36]. Work engagement is a direct reflection of an individual’s work performance and is a typical positive state in the workplace.

This study suggests that work engagement plays a mediating role between hierarchical plateau and taking charge behavior. Firstly, according to the JD-R model, an individual’s work motivation is triggered by abundant job resources. Job resources have an inherent motivational quality that can stimulate employees’ motivation, enhance work engagement, and thus positively impact work outcomes [37]. Empirical researches have also shown that job resources play a significant role in stimulating employees’ work engagement[38,39], as they not only help employees achieve their goals but also reduce the costs associated with meeting job demands [40]. Organizational support, as a type of job resource, can significantly enhance employees’ work engagement. However, a hierarchical plateau may lead civil servants to feel that they are not valued or supported by the organization and leadership [33]. On the other hand, career development opportunities are also considered an important job resource, and employees who perceive good development prospects tend to have higher levels of work engagement [41]. However, a hierarchical plateau implies a lack of promotion opportunities for civil servants, leading to a loss of resources associated with promotion, such as economic income, social status, and organizational power [15]. Moreover, according to the "resource loss spiral" effect of the COR theory, initial resource loss can trigger further resource loss [25]. Abd-Elrhaman et al [42] found that career plateau is highly negatively correlated with self-efficacy. Hobfoll [23]also suggests that employees stuck in a career plateau may lack self-discipline, self-esteem, or optimism, and are unable to focus on the positive aspects of work. In the meantime, according to the COR theory [25], being caught in a resource loss spiral can make individuals feel stressed and threatened. In order to compensate for the loss of resources, employees will activate psychological defense mechanisms to protect their remaining resources, leading to individuals disengaging from their work roles, which manifests as a decrease in work engagement [e.g.,^26^]. In short, when civil servants feel that their promotion space is limited and they are caught in a resource loss spiral, it weakens their vigor and enthusiasm for work, reducing work engagement.

Secondly, the low work engagement caused by a hierarchical plateau affects civil servants’ taking charge behavior. Taking charge is a constructive extra-role behavior that employees voluntarily and proactively undertake to improve existing work methods and organizational environments [1]. For employees, taking charge is a risky endeavor because it involves measures that break the routine, leading to uncertain outcomes or discomfort among organizational members, and may even cause conflicts [43]. Therefore, taking charge behavior requires not only certain knowledge and abilities but also a significant investment of time and energy, courage to face difficulties and setbacks, and the willingness to challenge the status quo. Work engagement, as a high-energy motivational state, not only stimulates individuals to invest a lot of energy and resources into the execution of their work roles but also encourages individuals to invest additional work resources into actions beyond the role [44]. Employees with this positive state usually devote more time and energy to their work, are full of vitality, dedicated, and actively seek to improve work methods, striving to overcome various difficulties encountered in work, thus providing important conditions for the formation of taking charge behavior [27–28]. Relevant research also highlights that when employees are highly engaged in their work, they tend to be more proactive [45]. The more employees are engaged in their work, the more motivated they are to take the initiative to change the current situation [46]. However, when civil servants exhibit low levels of work engagement, they are less likely to proactively initiate constructive change behavior. On one hand, low work engagement means that individuals lack passion and vitality in their work, thus having no extra energy to implement extra-role behavior. On the other hand, low work engagement indicates that individuals lack focus in their work, and with their attention scattered, it is difficult for them to notice the need for change, let alone bring about constructive change within the organization [27]. Furthermore, low work engagement also indicates a lack of dedication motivation, thus failing to inspire them to put in extra effort to implement organizationally constructive change behavior.

In summary, a hierarchical plateau may lead civil servants into a vicious cycle of the "resource loss spiral" effect, which will prompt civil servants to adopt a negative attitude towards work, reduce the level of work engagement, and thus have a negative impact on taking charge behavior. Thus, we hypothesize that:

H2: Work engagement mediates the relationship between hierarchical plateau and taking charge behavior.

### The moderating role of trait mindfulness

Mindfulness originates from Eastern Buddhism, with its original meaning being the lucidity and intentness of mind. In classical Buddhist concepts, mindfulness emphasizes awareness and attention to the present state [47]. Brown et al [48] defined mindfulness as “a receptive attention to and awareness of present events and experience,” a definition widely accepted by scholars in the management research field. Mindfulness can refer to both a state of consciousness and a personality trait or state-like trait [29]. Research suggests that mindfulness as a state-like trait has relatively low variability and a certain degree of plasticity [49]. In this study, we regard mindfulness as a positive personality trait [29] and, according to the COR theory and the JD-R perspective, consider it an important psychological resource. The present study speculates that trait mindfulness can mitigate the negative impact of hierarchical plateau on civil servants’ work engagement.

Firstly, trait mindfulness alleviates the stress perception of civil servants regarding hierarchical plateau. Brown and Ryan [29] found that individuals with high trait mindfulness experience less perceived stress. Individuals with high levels of trait mindfulness tend to focus more on the present and adopt a non-judgmental attitude when facing stress in their daily work, which makes them less affected by stress [50]. The mindful coping model also argues that highly mindful individuals actively re-evaluate and redefine stressors, reassessing the stress events to reduce the negative impact of stress [51]. Therefore, when facing the pressures of a hierarchical plateau, the negative impact on civil servants’ work engagement may be reduced due to the role of high trait mindfulness.

Secondly, trait mindfulness reduces the depletion of resources in individuals under hierarchical plateau conditions [52,53]. Individuals with high trait mindfulness possess stable attention and self-control abilities, enabling employees to focus more on current tasks [29]. Moreover, when facing negative events, individuals with high trait mindfulness adopt an open and inclusive attitude, accepting negative events without judgment, facing setbacks with equanimity, and actively seeking solutions [49]. In addition, the heightened focus and meta-awareness brought about by trait mindfulness can help employees gain mental space, reducing rumination and self-narrative judgment, thus liberating individuals from unconscious thoughts, habits, emotions, and unhealthy behavioral patterns, avoiding internal resource depletion [54]. Studies have confirmed that trait mindfulness has a positive impact on individuals’ psychological and behavioral aspects, such as improving sleep quality, enhancing cognitive flexibility, and reducing negative emotions [53]. Civil servants with high trait mindfulness are expected to suffer less resource loss and have a lower likelihood of falling into a resource loss spiral when facing a hierarchical plateau, thus having a smaller negative impact on work engagement.

Thirdly, trait mindfulness enhances an individual’s ability to acquire resources. According to the COR theory, individuals with abundant resources can acquire more resources [55] and generate more positive psychological states, competencies, and work behaviors [56]. Researchers have found that employee trait mindfulness helps individuals avoid resource depletion and enhance pleasant awareness and well-being experiences of the present through attention diversion and seeking alternative resources [57]; trait mindfulness can also enhance individual characteristic resources such as self-efficacy and optimistic resilience [58]. Furthermore, trait mindfulness can reduce the occurrence of work deviation behaviors and improve work capabilities by enhancing individuals’ focus and perception [59]. Therefore, civil servants with high trait mindfulness not only alleviate stress perception and reduce resource depletion when facing a hierarchical plateau but can also acquire new resources through different avenues. In contrast, civil servants with lower levels of trait mindfulness may find it difficult to objectively accept career dilemmas and respond positively when facing a hierarchical plateau, making them more likely to fall into a "resource loss spiral," affecting their level of work engagement. In this regard, we hypothesize that:

H3: Trait mindfulness plays a moderating role in the relationship between civil servants’ hierarchical plateau and work engagement. Specifically, when the level of civil servants’ trait mindfulness is high, the negative relationship between hierarchical plateau and work engagement is weaker, and vice versa.

Through the above analysis, employee work engagement mediates the negative relationship between hierarchical plateau and taking charge behavior of civil servants, while trait mindfulness moderates the negative relationship between hierarchical plateau and work engagement. Furthermore, this study posits that trait mindfulness will moderate the strength of the mediating effect of work engagement on the relationship between hierarchical plateau and taking charge behavior, in other words, this study proposes a moderated mediation hypothesis: under high trait mindfulness conditions, the negative impact of hierarchical plateau on civil servants’ work engagement will be weakened, and the indirect effect of hierarchical plateau on taking charge behavior through work engagement will also be weakened. Specifically, civil servants with high trait mindfulness are less affected by hierarchical plateau and will relatively consistently maintain their level of work engagement, thereby weakening the indirect effect of hierarchical plateau on employees’ taking charge behavior. In contrast, civil servants with low trait mindfulness will reduce their level of work engagement when facing a hierarchical plateau, enhancing the indirect effect of hierarchical plateau on taking charge behavior. Thus, the following hypothesis is proposed:

H4: Trait mindfulness moderates the mediating role of work engagement in the relationship between hierarchical plateau and taking charge behavior of civil servants, the higher the level of civil servants’ trait mindfulness, the smaller the negative effect of hierarchical plateau on civil servants’ taking charge behavior through work engagement, and vice versa.

**Fig 1:**
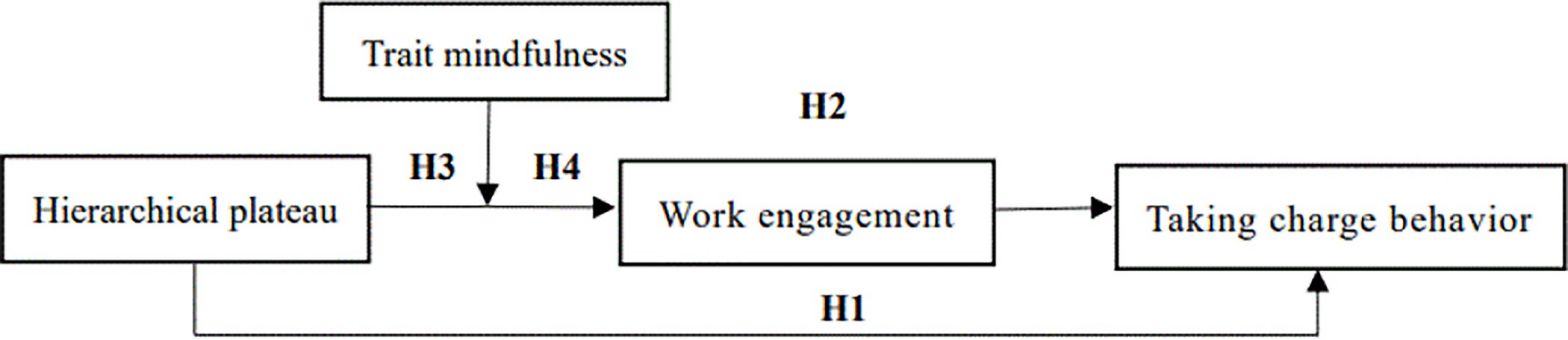
Theoretical model.

The theoretical model of this study is shown in Fig 1.

## Research methods

### Participants and procedure

The research participants were civil servants from the Pearl River Delta region in Guangdong Province, which is at the forefront of China’s reform and opening up. Using a snowball sampling method, with the consent of government officials and with their assistance, online survey questionnaires were distributed to their colleagues and, through them, to their peers. The questionnaires was used to collect information on participants’ hierarchical plateau, work engagement, taking charge behavior, trait mindfulness, and demographic characteristics. The cover letter attached to the questionnaire ensures respondents’ confidentiality and willingness to participate in the survey. The statement of the cover letter stated that if respondents agreed to be part of the survey, they were asked to participate; otherwise, they had the right to withdraw at any time, with specific details explained in the cover letter section of the survey. The Academic Committee Ethics Review Working Group of Beijing Institute of Technology, Zhuhai, approved this study (Approval No.: BITZH-BUS-2023-001), and since the cover letter ensured the respondents’ willingness to participate, the Ethics Review Working Group Consented that there was no need for any written formal consent from the respondents in this study. Data collection began on January 10, 2024, and ended on March 6, 2024.The questionnaires were distributed to the same group of participants in two stages through ’So Jump’. There was a 20-day interval between the first and second phases of the survey. The first stage collected information on participants’ demographic characteristics, hierarchical plateau, and trait mindfulness, while the second stage collected information on participants’ work engagement and taking charge behavior. A total of 307 matched questionnaires were received, and after excluding invalid questionnaires, 286 valid questionnaires remained.

In the valid sample, the proportion of men and women was almost equal (49.0% and 51.0%); the proportion of civil servants aged 25 and under was 44.8%, and those under 35 accounted for 77.7% of the total sample; those with less than 4 years of job tenure accounted for 50.0%, followed by those with 4–10 years of tenure, accounting for 36.4%; those with a bachelor’s degree or higher accounted for 77.3%; in terms of job category, integrated management category accounted for 31.5%, while professional and technical category accounted for 53.1%.

### Measures

The measurement of relevant variables is based on previously developed and well-established scales. Where necessary, the wording of some items has been adjusted.

#### Hierarchical plateau

Milliman [18] developed a career plateau scale that includes two dimensions: hierarchical plateau and job content plateau, consisting of 12 items. Allen et al [60] created a brief version of the career plateau scale based on Milliman’s scale, which includes 6 items for job content plateau and 4 items for hierarchical plateau. In this study, the hierarchical plateau is measured using the 4-item scale created by Allen et al [60]. The scale uses a 5-point Likert scale, scored from 1 (strongly disagree) to 5 (strongly agree). The Cronbach’s alpha coefficient for this scale is 0.843.

#### Work engagement

The Utrecht Work Engagement Scale (UWES-9) developed by Schaufeli et al. [61] is used. The scale includes three dimensions: vigor, dedication, and absorption, each with 3 items. This study measures it as a whole, using a 5-point Likert scale, ranging from 1 ("not at all") to 5 ("completely"). Sample items include: “At my work, I feel bursting with energy”. In this study, the Cronbach’s alpha coefficient for this scale is 0.922.

#### Taking charge behavior

Following the approach of Parker and Collins [62], the 4 items with the highest factor loadings from the Taking Charge Behavior Scale developed by Morrison and Phelps [1] are used to evaluate taking charge behavior of civil servants. The scale uses a 5-point Likert scale, scored from 1 (strongly disagree) to 5 (strongly agree), and is completed by the civil servants through self-report. The Cronbach’s alpha coefficient for this scale in this study is 0.850.

#### Trait mindfulness

The Mindful Attention Awareness Scale (MAAS) is used to assess the trait mindfulness of civil servants [29]. The scale consists of 15 items, using a 6-point Likert scale. All items are reverse-scored, so high scores represent high levels of trait mindfulness. The Cronbach’s alpha coefficient for this scale in this study is 0.964.

#### Control Variables

Drawing on existing research on taking charge behavior among civil servants [e.g., 3], gender, age, education, job tenure, and job category are controlled as the main variables affecting the proactive behavior of civil servants.

### Data analysis methods

This study first uses SPSS 19.0 for data preprocessing, correlation analysis, reliability testing, and common method bias testing; AMOS 17.0 software is used for confirmatory factor analysis; finally, SPSS is used for hierarchical regression and Process 3.0 plugin to test the research hypotheses.

## Results analysis

### Confirmatory factor analysis

Confirmatory factor analysis (CFA) was conducted on four research variables using AMOS 17.0 to examine and confirm the convergent validity and discriminant validity of each variable. The results of the CFA indicated that the average variance extracted (AVE) for each variable ranged from 0.570 to 0.641, which is above the threshold of 0.5; the composite reliability (CR) values fell between 0.843 and 0.964, exceeding the benchmark of 0.7, suggesting that the variables exhibit good convergent validity. Additionally, the discriminant validity of the variables was assessed through model comparison methods (see Table 1). The results presented in Table 1 demonstrate that the four-factor model not only met the recommended criteria (χ2 = 604.101, df = 458, χ2/df = 1.319, NFI = 0.910, TLI = 0.974, CFI = 0.976, RMSEA = 0.033) but also significantly outperformed the other five competing models. This confirms that the four variables measured in this study possess strong discriminant validity.

**Table 1:** Confirmatory factor analyses.

Model	χ2	df	χ2/df	Δχ2/Δdf	NFI	TLI	CFI	RMSEA
**Four-factor Model** **HP、WE、TC、TM**	604.101	458	1.319	/	0.910	0.974	0.976	0.033
**Three-factor Model** **HP+TC、WE、TM**	828.613	461	1.797	224.512***(3)	0.876	0.936	0.941	0.053
**Three-factor Model** **HP、TM、WE+TC**	978.725	461	2.123	374.624***(3)	0.854	0.910	0.916	0.063
**Two-factor Model HP+WE+TC、TM**	1270.968	463	2.745	666.867***(5)	0.810	0.860	0.870	0.078
**Three-factor Model** **HP+WE、TC+TM**	1416.491	463	3.157	812.39***(5)	0.782	0.827	0.782	0.087
**Single-factor Model** **HP + WE + TC+TM**	2705.486	464	5.831	2101.386***(6)	0.596	0.614	0.638	0.123
**Reference Value**	/	/	<3	/	>0.9	>0.9	>0.9	<0.08

Note. HP refers to hierarchical plateau; WE refers to work engagement; TC refers to taking charge behavior; TM refers to trait mindfulness.

### Common method variance test

Since all research data is provided by the respondents, there is a potential issue of common method bias. Therefore, it is necessary to control and test for this bias both procedurally and statistically. In terms of procedural controls: ①Some variables use a 5-point Likert scale, while others use a 6-point Likert scale; ② Some items are phrased positively, while others are negatively phrased. Additionally, during the survey administration, researcher emphasized the anonymity and confidentiality of the questionnaire and clarified that the data would be used solely for scientific research. In terms of methodological testing, the Harman’s single-factor test was employed to examine common method bias, using both exploratory factor analysis (EFA) and confirmatory factor analysis (CFA) for the assessment. The results demonstrated that the variance explained by a single factor was 39.38%, which is below the recommended threshold of 40% or 50%. In addition, the CFA results demonstrated in Table 1 show that the fit indices for the single-factor model were the poorest, and the fit indices for the four-factor model were significantly better than those for the single-factor model, suggesting that the common method bias in this study is not severe [63].

### Descriptive statistics

The means, standard deviations, and correlation coefficients among the variables are presented in Table 2. As shown in Table 2, hierarchical plateau is significantly negatively correlated with work engagement (r = -0.477, p<0.01), taking charge behavior (r = -0.525, p<0.01), and trait mindfulness (r = -0.446, p<0.01). Work engagement is significantly positively correlated with taking charge behavior (r = 0.447, p<0.01) and trait mindfulness (r = 0.348, p<0.01), and trait mindfulness is significantly positively correlated with taking charge behavior (r = 0.237, p<0.01).

**Table 2:** Means, standard deviations, and correlation coefficients of variables.

Variable	M	SD	1	2	3	4	5	6	7	8
**Gender**	0.490	0.501								
**Age**	1.937	1.116	0.018							
**Education**	2.976	0.810	-0.143**	-0.114*						
**Job tenure**	1.745	0.990	0.019	0.813**	-0.091					
**Job category**	2.224	0. 902	-0.034	0.091	0.022	0.013				
**Hierarchical plateau**	2.375	1.026	-0.094	0.039	0.062	0.053	0.014			
**Work engagement**	3.763	0.946	0.013	0.018	-0.029	-0.039	0.058	-0.477**		
**Taking charge**	3.790	0.992	0.090	-0.155*	-0.006	-0.129*	-0.080	-0.525**	0.447**	
**Trait mindfulness**	4.038	1.320	0.004	-0.001	-0.024	-0.052	0.016	-0.446**	0.348**	0.237**

Note

* p<0.1

** p<0.05.

### Hypothesis testing

This study employs the hierarchical regression method proposed by Baron and Kenny [64] to test the aforementioned hypotheses (Table 3). Initially, taking charge behavior is taken as the dependent variable, and control variables (i.e., gender, age, education, job tenure, and job category, the same below) are included in Model 5, followed by the addition of the independent variable hierarchical plateau in Model 6. The results demonstrate that hierarchical plateau has a significant negative impact on taking charge behavior of civil servants (β = -0.517, p< 0.001), supporting Hypothesis 1. Subsequently, control variables, hierarchical plateau, and work engagement are all included in the regression equation. Model 8 results show that work engagement has a significant positive impact on taking charge behavior (β = 0.271, p< 0.001), while the regression coefficient for hierarchical plateau on taking charge behavior changes from -0.517 to -0.387, remaining significant (p< 0.001), and the model’s explanatory power increases by 5.6%, significantly enhancing the explanatory effect of the regression equation. Therefore, work engagement plays a partial mediating role in the relationship between hierarchical plateau and taking charge behavior. Hypothesis 2 is verified. Furthermore, this study uses the Bootstrap method with 5,000 resampling iterations to further test the proposed mediating effect. The results demonstrate that the indirect effect of hierarchical plateau on taking charge behavior of civil servants through work engagement is significant (effect = -0.1263, standard error = 0.034, with a 95% confidence interval of [-0.1991, -0.0631], not including 0), providing further validation for Hypothesis 2.

**Table 3:** Hierarchical regression results.

Variable	Work engagement				Taking charge behavior			
	Model1	Model 2	Model 3	Model 4	Model 5	Model 6	Model 7	Model 8
**Gender**	0.011	-0.031	-0.025	-0.022	0.087	0.042	0.082	0.050
**Age**	0.131	0.128	0.110	0.102	-0.135	-0.139	-0.195*	-0.173*
**Education**	-0.027	-0.001	-0.001	0.011	-0.009	0.019	0.003	0.019
**Job tenure**	-0.149	-0.118	-0.099	-0.097	-0.020	0.013	0.048	0.045
**Job category**	0.049	0.054	0.052	0.061	-0.064	-0.058	-0.087	-0.073
**Hierarchical plateau**		-0.480***	-0.408***	-0.339***		-0.517***		-0.387***
**Trait mindfulness**			0.159**	0.186**				
**Hierarchical plateau × Trait mindfulness**				0.183**				
**Work engagement**							0.456***	0.271***
**R^2^**	0.012	0.239	0.259	0.288	0.036	0.299	0.242	0.339
**ΔR^2^**		0.227***	0.247***	0.029**		0.263***	0.206***	0.056***
**F**	0.676	14.579***	13.873***	14.015***	2.112	19.879***	14.854***	21.911***

Note

*** P<0.001

** P<0.01

* P<0.05;; All coefcients are standardized coefcients.

**Fig 2:**
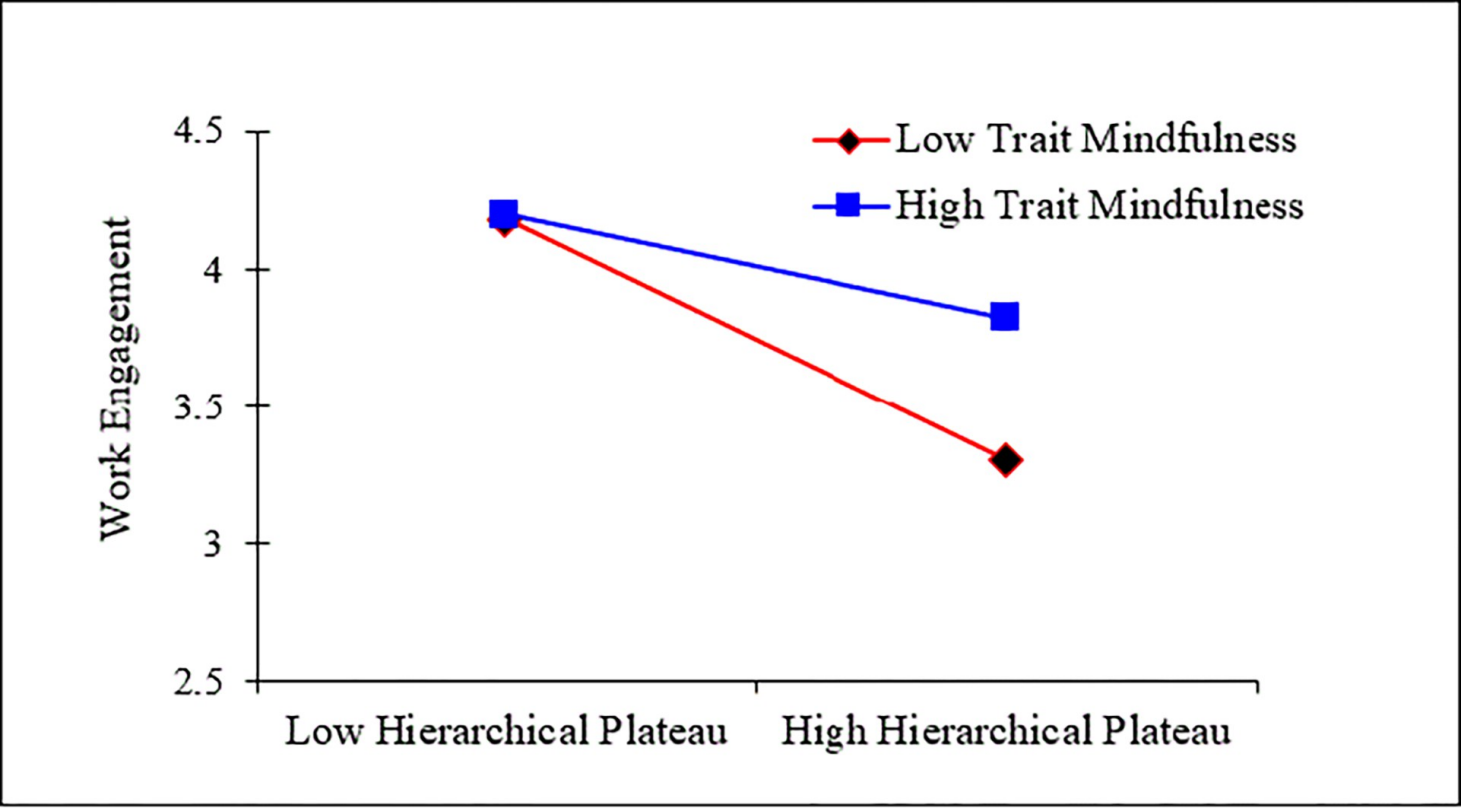
The moderating effect of trait mindfulness.

The moderating effect of trait mindfulness was examined using hierarchical regression analysis. The independent variable, hierarchical plateau, and the moderator variable, trait mindfulness, were centered, and their interaction term was calculated. Secondly, with work engagement as the dependent variable, control variables, hierarchical plateau, work engagement, and their interaction term were sequentially added. In Model 4 of Table 3, the interaction effect between hierarchical plateau and trait mindfulness positively affects work engagement (β = 0.183, p< 0.01), indicating that the higher the level of trait mindfulness among civil servants, the weaker the negative impact of hierarchical plateau on work engagement. Thus, Hypothesis 3 is supported. The collinearity diagnostics of the above hierarchical regression show that the tolerance values of all variables are greater than 0.1, and the VIF values are all less than 3, indicating that there is no significant multicollinearity problem among the variables. To more clearly present the moderating effect of trait mindfulness between hierarchical plateau and work engagement, following Aiken et al ’s [65] recommendation, trait mindfulness was divided into high and low levels by adding or subtracting one standard deviation from the mean, and an effect diagram was drawn to specifically analyze the interaction effect. As shown in Fig 2, the simple slope test results indicate that at low levels of trait mindfulness, hierarchical plateau significantly negatively affects work engagement, with a simple slope β = -0.473, SE = 0.060, t = -7.896, p<0.001, and the 95% confidence interval [-0.590, -0.355], which does not include 0; at high levels of trait mindfulness, the effect of hierarchical plateau on work engagement is not significant, with a simple slope β = -0.141, SE = 0.088, t = -1.608, p>0.05, and the 95% confidence interval [-0.312, 0.031], which includes 0.

Test of moderated mediation effect. The moderated mediation effect was examined using the Process plugin in SPSS, with a sample resampled 5,000 times and a 95% confidence interval. The results are shown in Table 4. The data indicate that at low levels of trait mindfulness, the indirect effect of work engagement is -0.1349, with a 95% confidence interval of [-0.2239, -0.0647], which does not include 0; at high levels of trait mindfulness, the indirect effect of work engagement is -0.0402, with a 95% confidence interval of [-0.0983, -0.0024], which does not include 0. This suggests that the mediating effect of work engagement is significant at different levels of trait mindfulness. In this situation, relying solely on the analysis of conditional mediation effects is not sufficient to conclude the existence of a moderated mediation effect. Therefore, Hayes [66] proposed a second method for testing conditional mediation effects, which is to use the index of moderated mediation to test for moderated mediation effects. Following this recommendation, the Index obtained from the Process calculation shows that the index of moderated mediation for the mediating effect of work engagement moderated by trait mindfulness is 0.0359, and the 95% confidence interval from the Bootstrap test is [0.0103, 0.0685], which does not include 0, indicating that the moderated mediation effect is significant, i.e., there is a significant difference in the mediating effect of work engagement at different levels of trait mindfulness. Thus, Hypothesis 4 is supported.

**Table 4:** Moderated mediation analyses.

Moderator Variable	The indirect effect of work engagement	SE	95% Confidence Interval	
			Lower Bound	Upper Bound
**Low trait mindfulness** **(-1SD)**	-0.1349	0.0410	-0.2239	-0.0647
**High trait mindfulness** **(+1SD)**	-0. 0402	0.0244	-0.0983	-0.0024

## Conclusion and discussion

### Conclusions

Based on the COR theory and the JD-R model, this paper examines the mechanism and boundary conditions of the impact of hierarchical plateau on taking charge behavior of civil servants, with work engagement as a mediating variable and trait mindfulness as a moderating variable. Analysis of 286 survey data reveals the following findings: ① hierarchical plateau negatively affects taking charge behavior of civil servants, with work engagement partially mediates this effect. ② trait mindfulness moderates the impact of hierarchical plateau on work engagement. ③ Under low trait mindfulness, the indirect effect of hierarchical plateau on taking charge behavior through work engagement is stronger compared to high trait mindfulness.

### Theoretical contributions

This study draws on the COR theory and the JD-R model to provide an explanatory framework for the factors that inhibit taking charge behavior among civil servants. The research reveals the relationship between the career development dilemmas of the civil servant group and their taking charge behavior, and deepens our understanding of how personality traits moderate the relationship between hierarchical plateau and taking charge behavior.

Firstly, the present study promotes an understanding of why civil servants resist taking charge. Based on the characteristics of career development within the civil servant group, this study expands the explanatory pathways for civil servant taking charge. Specifically, existing literature has largely explored the motivational factors for civil servants’ taking charge behavior, including public service motivation [10], transformational leadership (e.g., [9]), and fault-tolerant mechanisms [11,12], with less focus on the barriers to taking charge. Our study finds that hierarchical plateau, as a negative factor, suppresses taking charge behavior of civil servants. Therefore, this study offers a new perspective to understand the findings in the existing literature regarding civil servants’ taking charge behavior. Additionally, this paper responds to the call for strengthening research on the relationship between career plateau and individuals’ proactive behaviors [33]. Current research on the impact of career plateau on employee work performance has mostly focused on the relationship between career plateau and in-role performance, with less attention to the impact of career plateau on extra-role behaviors [33]. However, given the current, fast-changing work climate, employees are expected to extend their performance beyond their assigned duties [67]. For the public sector, the ability of civil servants to implement continuous bottom-up reforms and bring about constructive changes is also a goal and expectation [6]. This study enriches the literature on the relationship between career plateau and work performance through empirical analysis.

Secondly, The exploration of the mediating effect of work engagement in this study reveals the mechanism by which taking charge behavior occurs. Previous studies have more often regarded work engagement as a consequence of career plateau [e.g.,^26^,^68^]. In the study of the mechanism by which career plateau affect individuals’ attitudes and behaviors, the mediating effects of variables such as perceived organizational support [69] and job satisfaction [22] have been primarily explored. On the other hand, there is a lack of systematic research in academia on the impact mechanism of career plateau on employees’ taking charge behavior [22]. This study finds that work engagement is a key mediator in the relationship between hierarchical plateau and civil servants’ taking charge behavior. This supports the previously unverified hypothesis that this study is the first to link hierarchical plateau with a distal outcome through the mediating effect of work engagement. These findings are consistent with the core views of the COR theory and the JD-R model, in other words, individuals’ work motivation is triggered by abundant work resources, which then positively affect work outcomes [37]. Conversely, the loss of work resources can cause individuals to experience tension and stress [23,25], leading to employees’ negative attitudes towards work and ultimately producing outcomes that do not meet organizational expectations. Specifically, hierarchical plateau plunges civil servants into a spiral of resource loss, diminishing their vitality and enthusiasm for work; and work engagement, as an important antecedent of taking charge behavior[27,28], when civil servants to be under low levels of work engagement, they are less likely to proactively initiate constructive change behaviors. This study discusses and validates the mediating role of work engagement between hierarchical plateau and civil servants’ taking charge behaviors, providing new insights into the relationship between hierarchical plateau and taking charge behaviors.

Another contribution of this study is that it revealed the moderating role of trait mindfulness between hierarchical plateau and its outcomes, thereby enriching the existing literature. Based on the COR theory, individual traits, as initial resources, can play an initial resource effect, reducing the loss of individual resources and increasing the possibility of acquiring new resources [25]. The conclusions of this study are consistent with the logic of the COR theory, that is, the trait mindfulness of civil servants plays an initial resource effect, mitigating the negative impact of hierarchical plateau on work engagement, as well as the mediating effect of work engagement. Specifically, under high trait mindfulness, the impact of hierarchical plateau on work engagement is not significant, and the mediating effect of work engagement is also weaker. According to Fredrickson [70], work engagement promotes individuals to set higher, more challenging goals, and to build more enduring capabilities, helping individuals to take proactive actions. However, hierarchical plateau weakens individuals’ motivation for work engagement, which further affects taking charge behaviors of civil servants. In this process, trait mindfulness can serve as a crucial buffer against the erosion of work attitudes and behaviors by workplace stress. Previous studies have paid less attention to the moderating role of personality traits on the relationship between career plateau and related outcome variables. This study helps to more comprehensively understand how individual personality factors alleviate workplace stress, such as the impact of hierarchical plateau, by identifying trait mindfulness as an important boundary condition.

### Practical implications

First, since hierarchical plateau is a significant barrier that civil servants may encounter in their career development process, and this barrier can have negative effects on individual attitudes and behaviors [33], organizations should design multiple career development pathways for civil servants to avoid making "promotion" the sole criterion for measuring career success, and change the special and complex situation of "thousands of horses and soldiers rushing to official careers" in civil servant talent development. Organizations can develop personalized career development plans based on the individual characteristics, abilities, and needs. For example, by providing internal training, job rotation, cross-departmental collaboration, and lateral mobility, help civil servants enhance their capabilities and broaden their horizons. By providing a variety of career development opportunities, help them break through the bottlenecks in career development, stimulate the enthusiasm and positivity of civil servants, and thereby enhance their taking charge behavior.

Second, trait mindfulness, as a kind of trait-like variable, is influenced by both genetic and environmental factors, and appropriate education and intervention can improve individuals’ levels of mindfulness [49]. Therefore, the public sector should value the role of trait mindfulness in management practices through mindfulness training: on one hand, by improving civil servants’ awareness and attention levels through mindfulness training, enabling employees to efficiently focus on their current work and improve work efficiency; on the other hand, mindfulness training can also help employees regulate emotions, relieve stress, and enhance psychological resilience, making it easier for them to identify positive aspects of work and access organizational resources[53], thereby enhancing civil servants’ adaptability to the environment. In addition, organizations should also create a positive, open, and inclusive atmosphere, encouraging civil servants to be daring and proactive. By strengthening team building and enhancing organizational cohesion, enhance the sense of belonging and identity of civil servants, and stimulate their work enthusiasm and creativity.

### Limitations and directions for future research

This study has some shortcomings that need to be further improved in the future. Firstly, the sampling source is civil servants from the Pearl River Delta region in Guangdong, and the sample coverage is relatively limited, and the data obtained are all self-reported cross-sectional data. Although the research results confirm the hypotheses, they are not sufficient to strictly reflect the causal relationships between variables. Future research can expand the sample size and scope, adopt longitudinal research designs, and combine self-evaluation with peer evaluation to test the causal relationships between variables. Secondly, although more and more scholars regard mindfulness as a kind of trait-like variable, mindfulness can also be a state of consciousness [29]. Future research can start with state mindfulness, adopt experimental research methods, and conduct empirical research by collecting relevant data from experimental and control groups after employee mindfulness training. Thirdly, in this study, work engagement plays a partial mediating role in the process of hierarchical plateau affecting taking charge behavior, so there may be other mechanisms at play. To deepen our understanding of the mechanism of hierarchical plateau, future research can integrate different perspectives and comprehensively examine how hierarchical plateau affects the attitudes or behaviors of civil servants through different paths.

## Supporting information

S1 Dataset (XLSX)

S1 File (PDF)

S2 File (PDF)
