# Supplementary material for: The impact of hierarchical plateau on civil servants’ taking charge behavior: The role of work engagement and trait mindfulness
Source: PLoS One. 2024 Dec 17;19(12):e0315916. doi: 10.1371/journal.pone.0315916 (PMC11651623; doi:10.1371/journal.pone.0315916)
Supplement: S1 File — (PDF) [file pone.0315916.s002.pdf]

层级高原与公务员担当作为关系调查问卷（一）

亲爱的女士/先生:

您好！我们是来自北京理工大学珠海学院的研究人员。首先感谢您在百忙中参与此调查！本次调查旨在探讨层级高原对公务员主动担责行为的影响。本问卷仅为我们学术论文提供数据的整体分析，您的参与对我们如期完成研究目标至关重要。请您在认真阅读说明和题目后，根据自己的真实情况进行填写，答案并无对错之分。非常感谢您协助我们填写这份学术调查问卷。我们郑重承诺：您所提供信息仅限于学术研究之用，我们将严格保密，绝不外泄！再次感谢您的积极配合！

在正式填单问卷之前，请确认您是自愿且同意参与本次问卷调查！

□本人已知晓相关事项，自愿且同意参与本次问卷调查（在□内打√）

请填写您的姓氏首字母+手机后四位数字作为识别码（如 T6156）____________（仅供两期配对使用)。

第一部分：基本信息

本部分是个人基本资料，请您根据实际情况在相应的选项上打钩或填写

1.您的性别：□男 □女

2.您的年龄：□25 周岁及以下 □26-35 周岁 □36-45 周岁 □46-55 周岁 □55 周岁以上

3.您的教育程度：□高中/中专及以下 □大专 □本科 □硕士及以上

4.您的工作年限：□4 年以下 □5-10 年 □11-15 年 □16-20 年 □21 年以上

5.您的工作岗位：□综合管理类 □行政执法类 □专业技术类

第二部分

本部分是一些关于层级高原的描述，请根据您个人的感受，表达您对这些描述的同意程度，同意程度共分为五级，请选择符合您实际情况的选项。（1=非常不符合，2=比较不符合，3=不确定，4=比较符合，5=非常符合）

序号	题项	非常不符合	比较不符合	不确定	比较符合	非常符合
1	在目前的组织中，我升职的机会很有限	1	2	3	4	5
2	在目前的组织中，我被提拔的可能性很小	1	2	3	4	5
3	在目前的组织中，我已经升到了一个我难以再继续升职的岗位上	1	2	3	4	5
4	在目前的组织中，我不可能获得一个更高的职别或职称	1	2	3	4	5

第三部分

下面是一系列关于您日常生活经验的描述，请根据每一个陈述事件在您生活中发

生的频繁程度，共有 15 个题目，被分为 6 个等级，从 1-6 个数字中选出最符合实际经验的数字，注意请务必根据您的真实经验作答，而非您心中的頂期。请选择符合您实际情况的选项。（1=几乎从不；2=非常不频繁；3=有些不频繁；4=有些频繁；5=非常频繁；6=几乎总是）

序号	题项	几乎从不	非常不频繁	有些不频繁	有些频繁	非常频繁	几乎总是
1	有时我体验到一些情绪，过一会儿才会意识到这种情绪。	1	2	3	4	5	6
2	我会因为不小心、没注意或者想到其他事情而打碎物品或弄坏东西。	1	2	3	4	5	6
3	我发现静下心来关注当前发生的事情有些困难。	1	2	3	4	5	6
4	我前往要去的地方时，一路上对自己的走路行为或其他事物没有注意。	1	2	3	4	5	6
5	除非身体的紧张感或不舒适感引起我的注意，否则一般我都不会去关注身体的感觉。	1	2	3	4	5	6
6	如果我被第一次告知某个人的名字，我会很快地忘记这个名字。	1	2	3	4	5	6
7	我做事情好像是自动的过程，对于所做的事情没有太多觉知或者注意。	1	2	3	4	5	6
8	我匆匆做完一些事情而没有注意到这些事情本身。	1	2	3	4	5	6
9	我关注我想达到的目标，但我总是做与目标联系不大的事情。	1	2	3	4	5	6
10	我做工作或者任务是自动化的，不会去注意我在做什么。	1	2	3	4	5	6
11	我发现自己边听别人说话边做其他的事情。	1	2	3	4	5	6
12	我到达一个地方后会奇怪为什么我会来到这里。	1	2	3	4	5	6
13	我发现自己沉浸在对未来的幻想或者回忆过去的事情中。	1	2	3	4	5	6
14	我发现自己做事情时没有投入注意。	1	2	3	4	5	6
15	我吃零食的时候没有意识到自己正在吃东西。	1	2	3	4	5	6

问卷到此结束，感谢您的参与！

层级高原与公务员担当作为关系调查问卷（二）

亲爱的女士/先生:

您好！我们是来自北京理工大学珠海学院的研究人员。首先感谢您在百忙中协助完成此份问卷！本次调查是上次调查的延续。本问卷仅为我们学术论文提供数据的整体分析，您的参与对我们如期完成研究目标至关重要。请您在认真阅读说明和题目后，根据自己的真实情况进行填写，答案并无对错之分。非常感谢您协助我们填写这份学术调查问卷。我们郑重承诺：您所提供信息仅限于学术研究之用，我们将严格保密，绝不外泄！再次感谢您的积极配合！

在正式填单问卷之前，请确认您是自愿且同意参与本次问卷调查！

☐本人已知晓相关事项，自愿且同意参与本次问卷调查。（在☐内打√）

请填写您的姓氏首字母+手机后四位数字作为识别码（如 T6156）____________（仅供两期配对使用）

第一部分

本部分是个人基本资料，请您根据实际情况在相应的选项上打钩或填写

1.您的性别：☐男 ☐女

2.您的教育程度：☐高中/中专及以下 ☐大专 ☐本科 ☐硕士及以上

3.您的工作岗位：☐综合管理类 ☐行政执法类 ☐专业技术类

第二部分

请根据您个人的感受，表达您对这些描述的同意程度，同意程度共分为五级，请选择符合您实际情况的选项。（1=非常不符合，2=比较不符合，3=不确定，4=比较符合，5=非常符合）

序号	题项	非常不符合	比较不符合	不确定	比较符合	非常符合
1	在工作中，我感到自己迸发出能量	1	2	3	4	5
2	工作时，我感到自己强大并且充满活力	1	2	3	4	5
3	早上一起床，我就想要去工作	1	2	3	4	5
4	我对工作富有热情	1	2	3	4	5
5	工作激发了我的灵感	1	2	3	4	5
6	我为自己所从事的工作感到自豪	1	2	3	4	5
7	当工作紧张的时候，我会感到快乐	1	2	3	4	5
8	我沉浸于我的工作当中	1	2	3	4	5
9	我在工作时会达到忘我的境界	1	2	3	4	5

第三部分

本部分是对您日常工作行为的一些描述，请根据您个人的感受，表达您对这些描

述的同意程度，同意程度共分为五级，请选择符合您实际情况的选项。（1=非常不频繁，2=比较不频繁，3=不确定，4=比较频繁，5=非常频繁）

序号	题项	非常不频繁	比较不频繁	不确定	比较频繁	非常频繁
1	我经常尝试为单位或本部门引入改进的程序。	1	2	3	4	5
2	我经常尝试实施对组织更有效的新工作方法。	1	2	3	4	5
3	我经常尝试实施解决紧迫组织问题的解决方案。	1	2	3	4	5
4	我经常尝试引入新的结构、技术或方法来提高效率。	1	2	3	4	5

问卷到此结束，感谢您的参与！
