# Supplementary material for: The impact of hierarchical plateau on civil servants’ taking charge behavior: The role of work engagement and trait mindfulness
Source: PLoS One. 2024 Dec 17;19(12):e0315916. doi: 10.1371/journal.pone.0315916 (PMC11651623; doi:10.1371/journal.pone.0315916)
Supplement: S2 File — (PDF) [file pone.0315916.s003.pdf]

Survey on Factors Influencing the Taking Charge Behavior of Civil Servants (I)

Dear Madam/Sir,

Greetings! We are researchers from the Beijing Institute of Technology, Zhuhai. We would like to extend our heartfelt thanks for your participation in this survey despite your busy schedule! The purpose of this survey is to explore the factors that influence the taking charge behavior of civil servants. This questionnaire is solely for the overall data analysis of our academic paper, and your participation is crucial for us to meet our research objectives on time. After carefully reading the instructions and questions, please fill in the questionnaire based on your actual situation; there are no right or wrong answers. We greatly appreciate your assistance in completing this academic survey. We hereby promise: the information you provide will be used exclusively for academic research purposes, and we will keep it strictly confidential and never disclose it! Thank you again for your active cooperation!

□Before you officially start filling out the questionnaire, please confirm that you are participating voluntarily and consent to take part in this survey. **(Please tick the box '√'.)**

Please enter your first initial of your last name + the last four digits of your phone number as an identification code (e.g., T6156) __________ (for matching purposes between the two phases only).

Part I: Basic Information

This section contains personal basic information. Please tick the appropriate option or fill in the blanks according to your actual situation.

1.Gender：

□Male □Female

2.Age：

□under25years old □26-35 years old □36-45 years old □46-55 years old □above55 years old

3.Education：

□secondary school and below □college □undergraduate degree □master's degree and above

4.Tenure：

□less than 4 years □4-10yeas □11-15years □16-20 yeas □21 years and above

5. Job category

□Integrated management category □Administrative law enforcement category

□Professional and technical category

Part II

Please express your level of agreement with these statements based on your personal feelings.

The degree of agreement is divided into five levels. Please select the option that corresponds to your actual situation. (1 = Strongly Disagree, 2 = Disagree, 3 = Not Sure, 4 = Agree, 5 = Strongly Agree)

No.	Item	Strongly disagree	Disagree	Not Sure	Agree	Strongly agree
1	In the current organization, my chances of promotion are very limited.	1	2	3	4	5
2	In the current organization, the likelihood of my being promoted is very small.	1	2	3	4	5
3	In the current organization, I have reached a position from which it is difficult for me to be further promoted.	1	2	3	4	5
4	In the current organization, it is impossible for me to obtain a higher rank or title.	1	2	3	4	5

Part III

Here is a series of descriptions about your daily life experiences. Please rate the frequency with which each event occurs in your life based on the descriptions provided. There are 15 questions divided into 6 levels. Choose the number from 1 to 6 that best matches your actual experiences. Please ensure that your answers are based on your real experiences, not on your expectations. Select the option that corresponds to your actual situation. (1 = Almost Never; 2 = Very Seldom; 3 = Not Very Often; 4 = Fairly Often; 5 = Very Often; 6 = Almost Always)

No.	Item	Almost Never	Very Seldom	Not Very Often	Fairly Often	Very Often	Almost Always
1	I could be experiencing some emotion and not be conscious of it until some time later.	1	2	3	4	5	6
2	I break or spill things because of carelessness, not paying attention, or thinking of something else.	1	2	3	4	5	6
3	I find it difficult to stay focused on what's happening in the present.	1	2	3	4	5	6
4	I tend to walk quickly to get where I'm going without paying attention to what I experience along the way.	1	2	3	4	5	6
5	I tend not to notice feelings of physical tension or discomfort until they really grab my attention.	1	2	3	4	5	6

6	I forget a person's name almost as soon as I've been told it for the first time.	1	2	3	4	5	6
7	It seems I am "running on automatic" without much awareness of what I'm doing.	1	2	3	4	5	6
8	I rush through activities without being really attentive to them.	1	2	3	4	5	6
9	I get so focused on the goal I want to achieve that I lose touch with what I am doing right now to get there.	1	2	3	4	5	6
10	I do jobs or tasks automatically, without being aware of what I'm doing.	1	2	3	4	5	6
11	I find myself listening to someone with one ear, doing something else at the same time.	1	2	3	4	5	6
12	I drive places on"automatic pilot"and then wonder why I went there.	1	2	3	4	5	6
13	I find myself preoccupied with the future or the past.	1	2	3	4	5	6
14	I find myself doing things without paying attention.	1	2	3	4	5	6
15	I snack without being aware that I'm eating	1	2	3	4	5	6

The questionnaire ends here. Thank you for your participation!

Survey on Factors Influencing the Taking Charge Behavior of Civil Servants (II)

Dear Madam/Sir,

Greetings! We are researchers from the Beijing Institute of Technology, Zhuhai. We would like to extend our heartfelt thanks for your participation in this survey despite your busy schedule! This survey is a continuation of the previous one. This questionnaire is solely for the overall data analysis of our academic paper, and your participation is crucial for us to meet our research objectives on time. After carefully reading the instructions and questions, please fill in the questionnaire based on your actual situation; there are no right or wrong answers. We greatly appreciate your assistance in completing this academic survey. We hereby promise: the information you provide will be used exclusively for academic research purposes, and we will keep it strictly confidential and never disclose it! Thank you again for your active cooperation!

□Before you officially start filling out the questionnaire, please confirm that you are participating voluntarily and consent to take part in this survey. (Please tick the box '√'.)

Please enter your first initial of your last name + the last four digits of your phone number as an identification code (e.g., T6156) __________ (for matching purposes between the two phases only).

Part I: Basic Information

This section contains personal basic information. Please tick the appropriate option or fill in the blanks according to your actual situation.

1.Gender：

□Male □Female

2.Education：

□secondary school and below □college □undergraduate degree □master's degree and above

3. Job category

□Integrated management category □Administrative law enforcement category

□Professional and technical category

Part II

Please rate your level of agreement with the following statements based on your personal feelings. The level of agreement is divided into five levels. Please select the option that best matches your actual situation. (1 = Strongly Disagree, 2 = Disagree, 3 = Not Sure, 4 = Agree, 5 = Strongly Agree)

No.	Item	Strongly disagree	Disagree	Not Sure	Agree	Strongly agree
1	At my work, I feel bursting with energy	1	2	3	4	5
2	At my job, I feel strong and vigorous	1	2	3	4	5
3	When I get up in the morning, I feel like going to work	1	2	3	4	5
4	I am enthusiastic about my job	1	2	3	4	5
5	My job inspires me	1	2	3	4	5
6	I am proud of the work that I do	1	2	3	4	5
7	I feel happy when I am working intensely	1	2	3	4	5
8	I am immersed in my work	1	2	3	4	5
9	I get carried away when I am working	1	2	3	4	5

Part III

This section contains descriptions of your daily work behaviors. Please express your level of agreement with these descriptions based on your personal feelings. The level of agreement is divided into five levels. Please select the option that best matches your actual situation. (1 = Very Infrequently, 2 = Not Often, 3 = Not Sure, 4 = frequently, 5 = Very frequently)

No.	Item	Very Infrequently	Not Often	Not Sure	Frequently	Very frequently
1	I often tries to bring about improved procedures for the work unit or department.	1	2	3	4	5
2	I often tries to institute new work methods that are more effective for the organization.	1	2	3	4	5
3	I often tries to implement solutions to pressing organizational problems.	1	2	3	4	5
4	I often tries to introduce new structures, technologies, or approaches to improve efficiency.	1	2	3	4	5

The questionnaire ends here. Thank you for your participation!
